# miRNA-223 Suppresses Mouse Gallstone Formation by Targeting Key Transporters in Hepatobiliary Cholesterol Secretion Pathway

**DOI:** 10.7150/ijbs.65485

**Published:** 2021-10-25

**Authors:** Feng Zhao, Shiyu Ma, Yuling Zhou, Bailing Wei, Zhen Hao, Xiaolin Cui, Lina Xing, Gang Liu, Lingling Jin, Tonghui Ma, Lei Shi

**Affiliations:** 1 College of Basic Medical Sciences, Dalian Medical University, No. 9 West Section Lvshun South Road, Dalian 116044, China.; 2 Xiamen Cardiovascular Hospital, Xiamen University, Xiamen, 361004, China.

**Keywords:** miRNA-223, gallstone, ABCG5 and ABCG8

## Abstract

miRNA-223 has been previously reported to play an essential role in hepatic cholesterol homeostasis. However, its role in regulation of biliary cholesterol secretion and gallstone formation remains unknown. Hence, mice with conventional knockout (KO), hepatocyte-specific knockout (ΔHepa) / knockdown (KD) or gain expression of miRNA-223 were included in the study and were subjected to lithogenic diet (LD) for various weeks. The gall bladders and liver tissues were harvested for cholesterol crystal imaging, gallstone mass measurement and molecular analysis. Levels of cholesterol, bile salt, phospholipids, and triglyceride were determined in serum, liver tissues, and bile by enzyme color reactive assays. A 3' UTR reporter gene assay was used to verify the direct target genes for miRNA-223. LD-induced gallstone formation was remarkably accelerated in miRNA-223 KO, ΔHepa, and KD mice with concurrent enhancement in total cholesterol levels in liver tissues and bile. Key biliary cholesterol transporters ABCG5 and ABCG8 were identified as direct targets of miRNA-223. Reversely, AAV-mediated hepatocyte-specific miRNA-223 overexpression prevented gallstone progression with reduced targets expression. Therefore, the present study demonstrates a novel role of miRNA-223 in the gallstone formation by targeting ABCG5 and ABCG8 and elevating miRNA-223 would be a potentially novel approach to overcome the sternness of cholesterol gallstone disease.

## Introduction

Gallstone is a disease of the digestive system with high incidence and approximately 10-20% prevalence in developed countries 1 with a gradual increase. The increasing annual cholecystectomy cases incur a huge economic stressand public health-care burden 2. Based on chemical composition, cholesterol gallstone counts of 80-90% gallstone cases 2, 3 that appears due to imbalanced hepatic cholesterol metabolism associated with metabolic syndromes such as type 2 diabetes, obesity, hyperlipidemia, and high caloric intake. Dysregulation in genes controlling hepatobiliarycholesterol secretion has been shown to have a remarkable influence on cholesterol gallstone development. ABCG5 and ABCG8 are two heterodimeric ATP-binding cassette (ABC) transporters on hepatic canalicular membrane and are major contributors of hepatocytes cholesterol secretion into bile 4, as evidenced by approximately 75% reduced biliary cholesterol secretion rate in *Abcg5* and/or *Abcg8* KO mice 5. A genome-wide association study (GWAS) revealed that genetic *ABCG5/8 loci* mutations are closely associated with gallstone diseases 1. In* Abcg5/8* KO mice, LD-induced gallstone formation was greatly attenuated 6. Other than ABCG5/8, scavenger receptor class B type I (SR-BI), coding by *Scarb1* in mice, has also been identified for efficient conduction of biliary cholesterol secretion in physical condition 7. Adenovirus-mediated hepatic overexpression of SR-BI has preferentially increased its canalicular membranes distribution and resulted in accelerated biliary cholesterol secretion rate in wild type (WT) or* Abcg5* KO mice 8. In contrary,* Scarb1* KO or* Scarb1/Abcg 5* double KO mice exhibited reduced biliary cholesterol secretion 5. Besides, SR-BI on hepatocyte basolateral membrane is well accepeted to conduct the cholesterol uptake from circulating HDL that plays a key role in maintaining serum cholesterol homeostasis 8, 9. Therefore, these hepatic cholesterol transporters constitute an efficient cholesterol biliary secretion pathway and are proposed to be potential therapeutic targets for gallstone diseases.

The miRNAs play an important role in modulating pathophysiological processes in a fine-tuning manner by suppressing their target mRNA translation. In the liver, miRNAs are reported to actively contribute to metabolic homeostasis of glucose 10, 11, lipids as well as cholesterol 12, 13. miRNA-223 gene is localized on the X chromosome and was initially identified as a hematopoietic specific miRNA that manipulates granulocyte differentiation 14, macrophage phenotype transition in the context of obesity 15, 16, and platelets ultra-activation under the diabetic situation 17. As to specific liver pathological events, miRNA-223 was able to prevent APAP-induced liver damage via modulating neutrophil-mediated death-associated molecular pattern (DAMP) 18. Moreover, miRNA-223 limits the neutrophil oxidative stress through suppressing IL-6-p47^phox^ pathway and protects the liver from alcoholic injury 19. Notably, Vickers* et al.* 13 reported that elevated intracellular cholesterol increases hepatocyte miRNA-223 expression and influences hepatic cholesterol homeostasis by different mechanisms *i.e.* attenuating hepatocyte cholesterol uptake from blood by targeting SR-BI expression, suppressing cholesterols synthesis via targeting HMGCS1 and SC4MOL, and promoting cholesterol efflux by indirectly upregulating basolateral ABCA1 expression in hepatocytes. Given that, we further wonder whether or not miRNA-223 also influences biliary cholesterol secretion pathway as well as consequential gallstone formation.

The purpose of the present study is to determine the significance of hepatocyte miRNA-223 in gallstone pathogenesis with a special focus on its role in regulating the expression of key transporters in the biliary cholesterol secretion pathway.

## Materials and methods

### Animals

miRNA-223 KO and conditional KO mice (cKO) were generated in C57BL/6J line by Shanghai Biomodel Organism Science & Technology Development Co., Ltd. Conventional, hepatocyte-specific, and myeloid-specific miRNA-223 KO mice were achieved by crossbreeding miRNA-223 cKO with Alb-Cre^Tg^ and Lyz2-Cre^Tg^ mouse lines provided by Biomodel Organism Science & Technology Development Co., Ltd.

Adeno-Associated Viruses (AAVs) used in this study were purchased from Hanbio Biotechnology Co., Ltd. For hepatocyte-specific knockdown of miRNA-223 expression, AAV8-TBG-Flag-Cre-T2A-GFP (1×10^11^ virus genome) or AAV8-TBG-GFP (as control, 1×10^11^ virus genome) was injected intravenously via tail vein into miRNA-223 cKO mice. For hepatocyte-specific overexpression of mouse miRNA-223 precursor sequence, wild-type (WT) mice were received a one-time injection of AAV8-U6-miRNA-223 precursor/CMV-GFP or AAV8-CMV-GFP (each 1×10^11^ virus genome) via the tail vein.

### Murine Gallstone Model

The 8 weeks old male mice with C57BL/6J genetic background were used in the study and fed with lithogenic diet (TP2890, containing 15% lard fat, 1.25% cholesterol, and 0.5% sodium cholic acid) or chow (LAD0011) purchased from TROPHIC Animal Feed High Tech Co. Ltd, China for the indicated time periods.

### Bile Flow Rate Measurements

The procedure was as described in previous publication 20. Briefly, male mice were fasted for 6 h allowing free access to water. After ligating the cystic duct, the common bile duct was cannulated with PE-10, and hepatic bile was collected for 30 min to determine the flow rate. During surgery and hepatic bile collection, the mice were under anesthesia with isoflurane and maintained at 37 °C on a hot plate.

### Study Approval

Animals were maintained in Dalian Medical University Laboratory Animal Center under the specific pathogen-free condition, and all animal study procedures were approved (#AEE17036) by the Ethics Committee for Biology and Medical Science of Dalian Medical University.

### Cell Culture

Mouse primary hepatocytes were isolated by gradient density centrifugation of 30% Percoll solution after liver perfusion using Collagenase IV (Sigma). Primary hepatocytes were cultured in William's E Medium (Thermo Fisher Scientific) supplemented with 10% fetal bovine serum, 1% ITS (Sigma), 2 mM L-glutamine, and 100 nM dexamethasone. HEK293T cells were maintained in Dulbecco's Modified Eagle's Medium supplemented with 10% fetal bovine serum plus penicillin (100 mg/mL) and streptomycin (100 mg/mL). Cells were kept in a 5% CO_2_ and 95% air humidified incubator at 37 °C. miRNA-223 mimics and negative control (GenePharma, China) were transfected into primary hepatocytes via Lipofectamine RNAi MAX Transfection Reagent (Thermo Fisher Scientific) according to the manufacturer's instructions.

### FACS analysis

After Collagenase IV *in vivo* digestion, hepatic cell suspension or purified hepatocytes were incubated with indicted primary antibodies at room temperature for 20 min in the dark and different cell populations were sorted and further analyzed by MOFLO ASTRIOS^EQ^, BECKMAN COULTER. The liver cell mixtures was firstly scattered by FSC/SSC, and signal cells population was gated by FSC-A/FSC-H, living cells population was further gated by DAPI negative selection, and the CD11b+/Gr1+ positive cell percentages were determined from those “living cell” population.

### Plasmids and Antibodies

The partial 3' UTR regions of mouse *Abcg5* (Genebank: NM_031884.2, Range 2095 to 2411) and *Abcg8* (Genebank: NM_026180.3, Range 2394 to 2772) were obtained by RT-PCR from liver tissue cDNA and subcloned into the pMIR-Reporter plasmid. The mutants for miRNA-223 binding sequences in 3' UTRs of *Abcg5* and *Abcg8* were obtained by using the Fast Mutagenesis System (TRANSGEN BIOTECH). The primer sequences for cloning and mutation are all shown in Supplementary Table 1. The antibodies usage and information are presented in Supplementary Table 2.

### Biochemical Analysis

Total cholesterol (T-Chol. Catalog No. A111-1-1), high-density lipoprotein cholesterol (HDL-C Catalog no. A112-1-1), low-density lipoprotein cholesterol (LDL-C Catalog No. A113-1-1), total bile acid (TBA Catalog No. E003-2-1), and triglycerides (TG Catalog No. A110-1-1) concentrations were analyzed using Kits (Nanjing JIANCHENG Bioengenering, Najing, China) according to the manufacturer's instructions. Phospholipid (PL) concentrations were quantified using Wako Kits (Catalog No. 296-63801, Osaka, Japan) according to the manufacturer's instructions. The activities were measured using the Multi-Mode Microplate Reader (BioTek Synergy NEO). Bile cholesterol saturation index was calculated as described in the previous report 21.

### Bile Cholesterol Crystal Analysis and Gallstone Examinations

The contents of gallbladders were placed onto glass slides or centrifugal tubes after cholecystectomy, followed by measuring the weights of gallstone after removal of bile and dry by air. Bile cholesterol crystal was evaluated under polarized-light microscopy (Olympus BX63) and the crystal type was classified according to previous study 22, 23. Gallstone and KBr mixture was prepared at a ratio of 1:150 and the contents of cholesterol, bile acid, and phospholipid were further analyzed by Fourier Infrared Spectrograph (Nicolet 6700 FlexFT-IR SPECTROMETER, Thermo Fisher Scientific).

### Histological Analysis

After scarification, animal livers were embedded in paraffin or optimal cutting temperature compound (OCT). Paraffin-embedded tissue sections were stained with hematoxylin and eosin (H&E) for morphological studies. OCT-embedded tissue cryosections were incubated with rhodamine-phalloidin (Sigma) and DAPI for fluorescence analysis.

### Real-Time quantitative PCR and Genotyping

Total RNA was extracted from mouse liver tissues using the TRIzol (Invitrogen) according to the manufacturer's instructions and cDNAs were reverse-transcribed using the FastKing RT Kit (TIANGEN). The cDNA samples were amplified by Real-time quantitative PCR (RT-qPCR) using SYBR Green qPCR Master Mix (Bimake) and *18S* was used as a standard reference. For genotyping, the genomic DNA from indicated tissues or primary cells were prepared by TIANamp Genomic DNA Kit (TIANGEN) and further identified by PCR using specific primers. The primer sequences used in this study are presented in Supplementary Table 1.

### Western Blot

Mouse tissues or primary hepatocytes homogenates were subjected to SDS-PAGE, transferred to nitrocellulose (NC) membranes, and incubated with specific primary antibody. After washing, the membranes were incubated with anti-mouse Dylight 680 or anti-rabbit Dylight 800 secondary antibody (Abbkine). The membranes were then scanned using the Odyssey CLx Imaging System (LICOR), and the images were generated employing the Image Studio software.

### Luciferase Activity Assay

HEK293T cells (5 × 10^5^/well) were cultured in 24-well plates one day before transfection, and then co-transfected with 60 ng 3′-UTR luciferase reporter plasmids, 20 ng β-galactosidase plasmids, 20 nM miRNA-223 mimics or the negative controls by using Lipofectamine RNAi MAX Transfection Reagent (Thermo Fisher Scientific) according to the manufacturer's instructions. Cells were harvested 24 h post-transfection and further subjected to luciferase and β-galactosidase activitydetection by using the D-Luciferin (BD Biosciences) and o-nitrophenyl-β-d-galactoside (ONPG), respectively. The firefly luciferase and β-galactosidase activities were measured using the Multi-Mode Microplate Reader (BioTek Synergy NEO). The results were normalized as the ratio of firefly luciferase activities to β-galactosidase activities.

### Statistical Analysis

Data are expressed as the mean ± S.E.M, and statistical evaluation was performed using Student's t-test for unpaired data. The values of *p*<0.05 or less were considered statistically significant.

## Results

### Hepatocytes miRNA-223 Expression is increased with LD-induced Gallstone Formation

**Figure 1:**
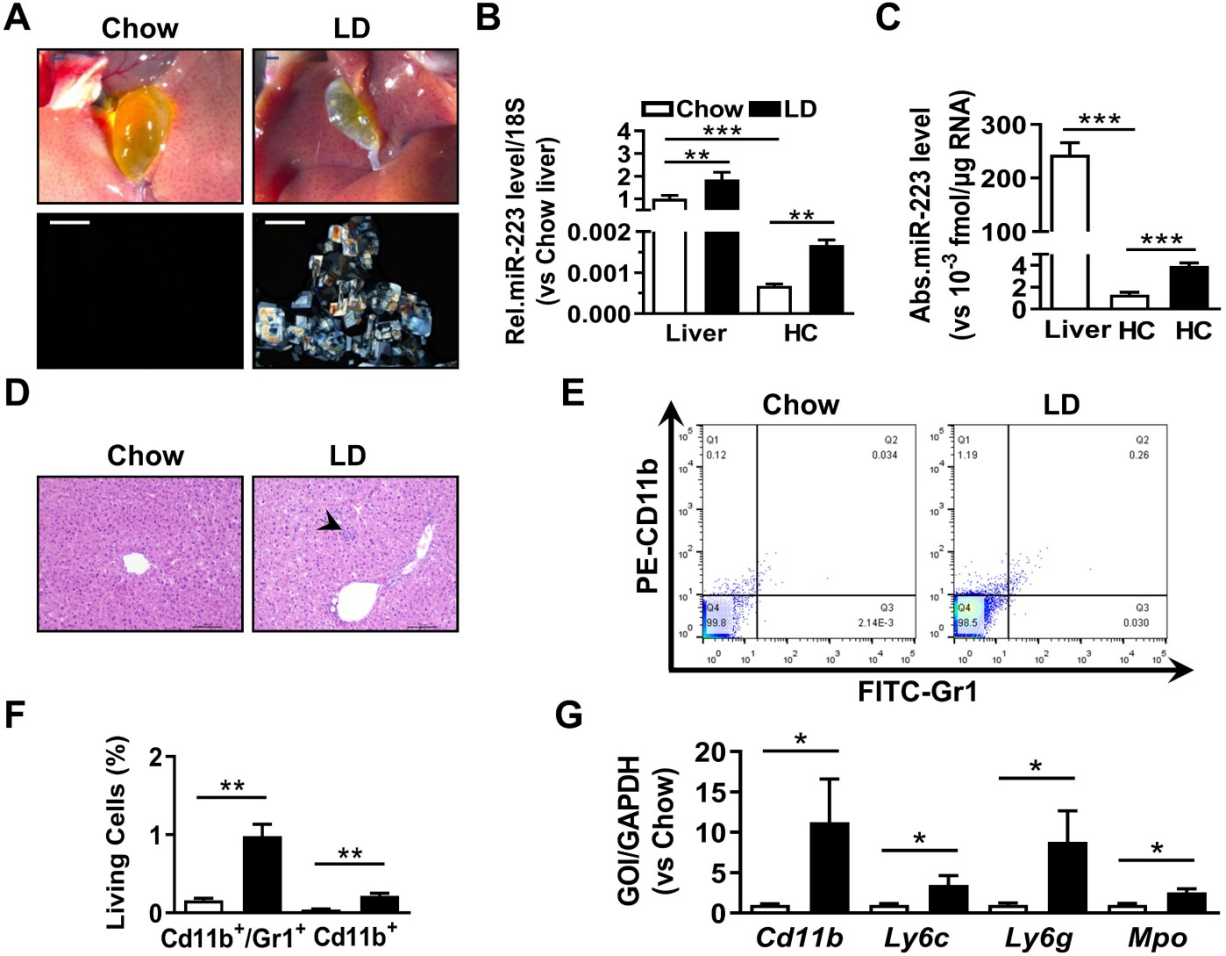
** The expressional alternation of miRNA-223 in mouse liver during gallstone progression.** WT male mice with 8-10 weeks were fed for lithogenic diet (LD) or chow diet for 5 weeks. **(A)** Representative images showing the gall bladders and cholesterol crystals in bile. Scale bars, 1 mm (gall bladder images) and 250 µm (cholesterol crystal images); RT-qPCR detecting (**B**) the relative or (**C**) absolute miRNA-223 expression in liver tissues and primary hepatocytes (HC) from chow and LD fed mice with 18s as reference gene (n=5-6 mice per group). **(D)** H&E staining for liver sections. Scale bars, 100 µm. **(E&F)** FACS analysis for hepatic infiltrating CD11b^+^/Gr1^+^ cell population (n=3 mice per group). **(G)** Leucocytes marker genes were assessed by RT-qPCR (n=3 mice per group); *p<0.05; **p<0.005; ***p<0.001.

To analyze the correlation of hepatic miRNA-223 expression with cholesterol gallstone formation, liver tissues and primary hepatocytes were analyzed from wildtype mice on chow or challenged with lithogenic diet (LD) for 5 weeks. Along with LD-induced gallstone generation (Figure 1A) miRNA-223 expression (miRNA-223-3p levels, as its other mature form miRNA-223-5p is undetectable in the livers) was significantly increased in both liver tissues and freshly isolated primary hepatocytes (HCs) (Figure 1B-C), although the absolute miRNA-223 expression level in primary hepatocytes only accounts for ~0.07% of that in liver tissues (Figure 1C). Besides, a slightly increased hepatic leukocytes infiltration in LD-treated livers was observed (as indicated by arrow shown in Figure 1D), which was further evidenced by increased hepatic Gr1/CD11b-positive cell population (Figure 1E-F) and mRNA expression of leukocytes markers* CD11b, Ly6g, Ly6c* and *Mpo* (Figure 1G). Therefore, it is suggested that hepatic nonparenchymal cells especially the infiltrating leukocytes may account for the increased miRNA-223 levels in LD-treated livers as miRNA-223 was known to highly expressing in leukocytes 14.

### miRNA-223 Knockout Accelerates LD-induced Gallstone Formation in Mice

**Figure 2:**
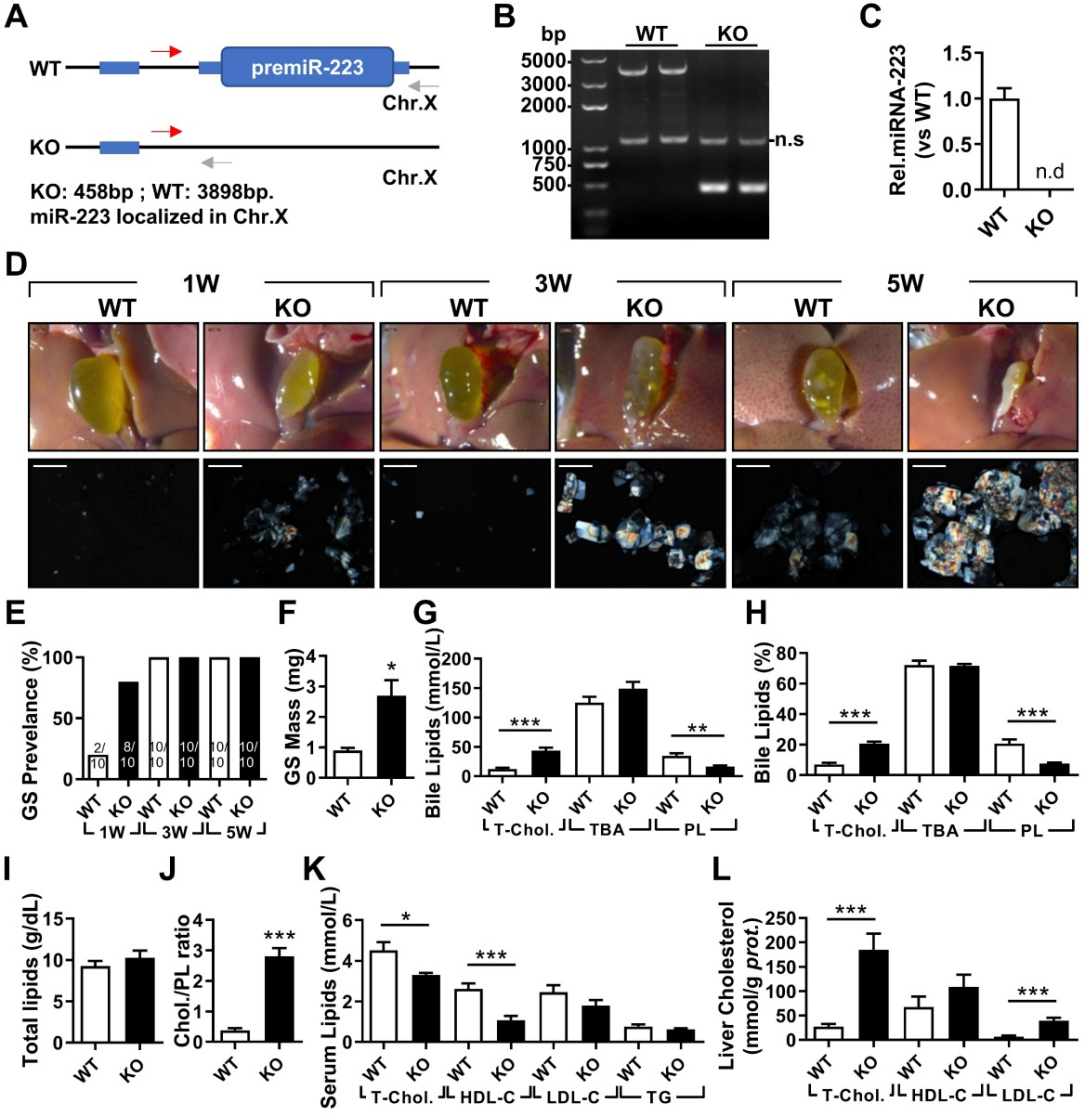
** The effects of miRNA-223 KO on mice gallstone formation.** miRNA-223 KO and WT male littermates were subjected to LD feeding for 1, 3 and 5 weeks, and in the indicated time points, livers, serum and bile were harvested after overnight starvation for further detection. **(A & B)** Cartoon showing the miRNA-223 KO detection method by genomic DNA PCR, miRNA-223 is localized in X chromosome, WT: 3898bp, KO: 458bp, ns: non-specific bind. **(C)** RT-qPCR detecting miRNA-223 expression in livers, n=5 mice each group. **(D)** Representative images showing the gall bladders and cholesterol crystals in bile. Scale bars, 1 mm (gall bladder images) and 250 µm (cholesterol crystal images). **(E)** Gallstone prevalence with LD feeding summarized from 10 animals from each group; **(F)** weighted gallstone mass after 5 weeks LD treatment (n=5 mice per group); **(G & H)** bile lipids content and percentage; **(I)** total bile lipids contents; **(J)** ratio of cholesterol to PL in bile ( n=6-8 mice per group); biochemistry parameters of Cholesterol, HDL-C,LDL-C and TG were separately determined in **(K)** serum and **(L)** liver tissues (n=10 mice per group). *p<0.05; **p<0.005; ***p<0.001versus WT.

To determine the importance of miRNA-223 in gallstone formation, we firstly generated miRNA-223 conditional KO mice (miRNA-223 cKO, Supplementary Figure 1A) and further obtained conventional miRNA-223 KO micevia crossbreeding miRNA-223 cKO mice with EIIA-Cre^Tg^ mice (Supplementary Figure 1B). KO was examined for the removal of miRNA-223 loci in liver genomic DNA (Figure 2A-B), which was further comfirmed by undetectable miRNA-223 expression in livers (Figure 2C). As shown in Figure 2D, KO mice accelerated gallstone progression with more agglomerated cholesterol monohydrate crystals in the bile than that in WT littermates. It was further evidenced by a greater gallstone forming rate one week post LD feeding (80% in KO *vs.* 20% in WT, as given in Figure 2E) and increased gallstome mass after 5 weeks LD treatment (2.70 ± 0.85 mg in KO *versus.* 0.90 ± 0.12 mg in WT, as shown in Figure 2F). However, the mice of both genotypes represented a comparable body growth curve, fat, muscle contents (Supplementary Figure 2A-D) as well as fasting serum lipids levels of total cholesterol (T-Chol.) and triglyceride (TG) (Supplementary Figure 2E).

### miRNA-223 Knockout Results in Cholesterol Super-Saturation in the Bile

In the bile, KO mice exhibited significantly elevated levels of T-Chol. and reduced phospholipids (PL) content with no change in levels of total bile acid (TBA) (Figure 2G), resulting in a relative higher portion of cholesterol (20.67% in KO vs 7.04% in WT, as given in Figure 2H) and lower portion of PL (7.66% in KO vs 20.70% in WT, as presented in Figure 2H). Although there was no difference in total biliary lipids contents (Figure 2I), a remarkable elevationin of cholesterol/PL ratio in KO mice were discovered (Figure 2J), indicating a super-saturation status for cholesterol. In parallel, significantly reduced serum T-Chol. and HDL-C (Figure 2K), enhanced liver T-Chol. and LDL-C levels (Figure 2L) and comparable liver TG contents (Supplementary Figure 2F) were also observed in KO mice. There was no obvious liver injury as indicated by similar serum ALT and AST activities between the both KO and WT mice (Supplementary Figure 2G), but a slight increase in infiltrating inflammatory cells was observed in KO livers (Supplementary Figure 2H).

### Myeloid-specific miRNA-223 KO Has No Effect on Mouse Gallstone Formation

Myeloid miRNA-223 has been reported to profoundly influence cholesterol and TG metabolism in macrophages 13, 24 and hepatic immunoregulation 19, 25. To rule out the possible involvement of myeloid miRNA-223 in cholesterol gallstone development, myeloid-specific miRNA-223 KO mice (ΔMye) were generated by crossbreeding the miRNA-223 cKO with Lyz-Cre^Tg^ mice (Supplementary Figure 1B). Precise genomic excision for miRNA-223 loci resulted in ~98% reduction of miRNA-223 expression in ΔMye bone marrows (Supplementary Figure 3A & B). In ΔMye liver, we detected a dramatic decrease (~78%) of miRNA-223 expression than that in cKO mice (Supplementary Figure 3B), suggesting that the Lyz2-positive nonparenchymal cells contribute to a major portion of general hepatic miRNA-223 content. There was no obvious difference in major gallstone phenotypes (Supplementary Figure 3E-F) and liver injury (Supplementary Figure 3D), although a slightly lower levels of T-Chol. and LDL-C were detected in ΔMye serum (Supplementary Figure 3C).

### Hepatocyte-specific Depletion of miRNA-223 Increases Gallstone Formation

**Figure 3:**
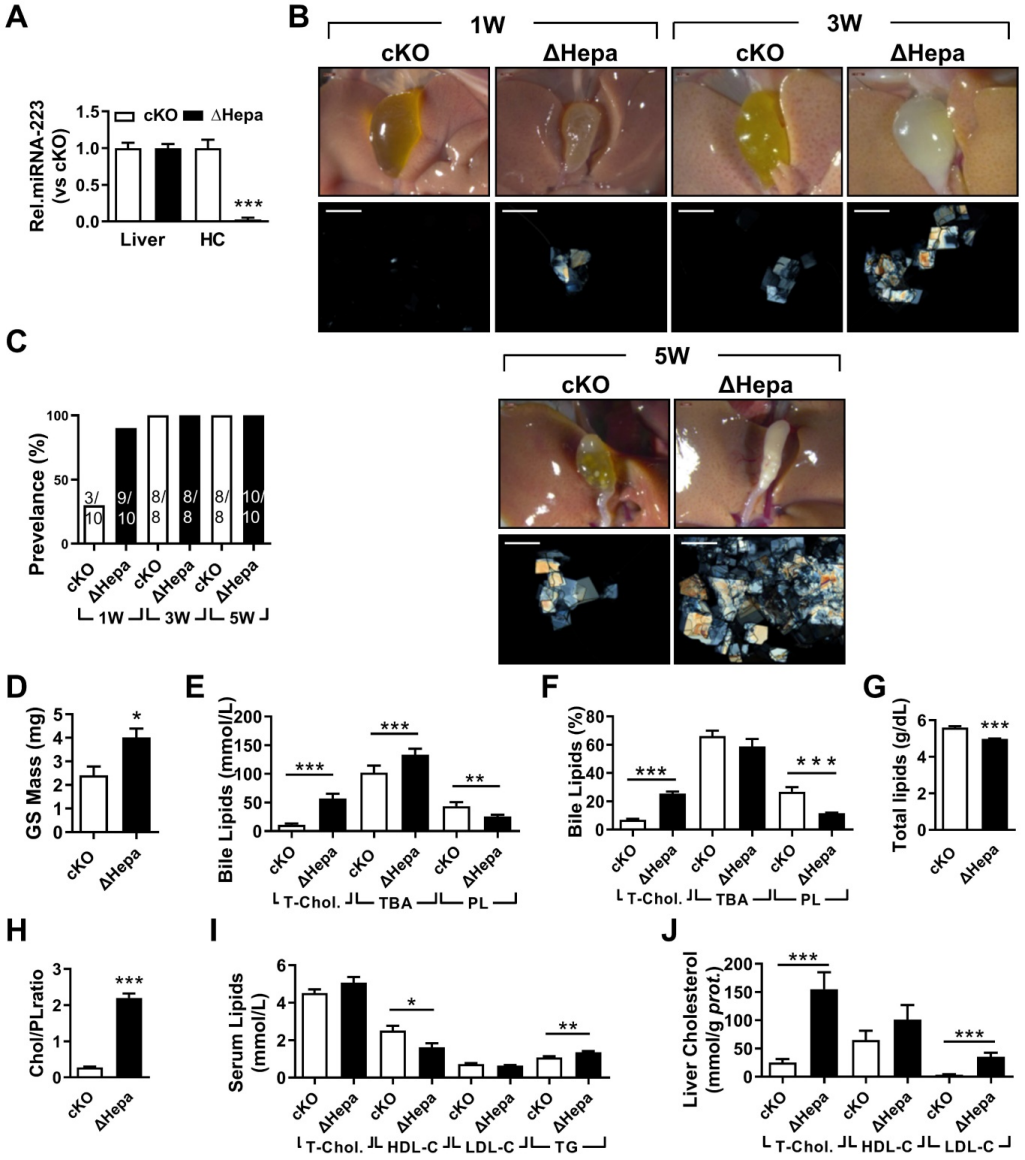
** The influences of hepatocytes specific miRNA-223 KO on mice gallstone formation.** Hepatic specific miRNA-223 KO (ΔHepa) and cKO male littermates were subjected to LD feeding for 1 to 5 weeks, and in the indicated time points, livers, serum and bile were harvested after overnight starvation for further detection. **(A)** RT-qPCR showing miRNA-223 expression in livers or primary hepatocytes (n=5 mice each group). **(B)** Representative images showing the gall bladders and cholesterol crystals in bile. Scale bars, 1 mm (gall bladder images) and 250 µm (cholesterol crystal images). **(C)** Gallstone prevalence with LD feeding (n=8-10 mice each group). **(D)** Weighted gallstone mass (n=4 mice each group). **(E & F)** Bile lipids content and percentage; **(G)** total bile lipids contents; **(H)** ratio of cholesterol to PL in bile (n=8-10 mice each group); biochemistry parameters of Cholesterol, HDL-C, LDL-C and TG were separately determined in **(I)** serum and **(J)** liver tissues (n=8-10 mice each group); *p<0.05; **p<0.005; ***p<0.001versus cKO.

To clarify the importance of hepatocytes miRNA-223 in LD-induced gallstone development, we generated hepatocyte-specific miRNA-223 KO mice (ΔHepa) by crossbreeding the cKO mice with Alb-Cre^Tg^ mice (Supplementary Figure 1B). Interestingly, there was an approximately 97.35% decreased miRNA-223 expression was assessed in freshly isolated primary ΔHepa hepatocytes compared to that in cKO hepatocytes, whereras comparable levels of miRNA-223 were detected in livers tissues of both genotypes (Figure 3A). Upon five weeks LD treatment, ΔHepa remarkably promoted gallstone progression (Figure 3B). As early as one week after LD feeding, ΔHepa displayed a faster gallstone formation rate (90% in ΔHepa *vs*. 30% cKO, as given in Figure 3C), with a significant increase in gallstone mass (4.02±0.37 mg in ΔHepa *vs*. 2.4±0.38 mg in cKO, as presented in Figure 3D). Similarly, ΔHepa mice showed the alternations of lipids contents in bile (Figure 3E-H), serum (Figure 3I), liver tissue (Figure 3J, Supplementary Figure 4E) were greatly consistent with that appeared in KO mice, except reduced biliary total lipids (Figure 3G) and comparable T-Chol. and elevated TG levels in serum (Figure 3J). Moreover, ΔHepa would not cause further liver damage and inflammatory cells infiltration (Supplementary Figure 4A & C).

### AAV-Cre mediated Hepatic Knockdown of miRNA-223 Accelerates Gallstone Formation

**Figure 4:**
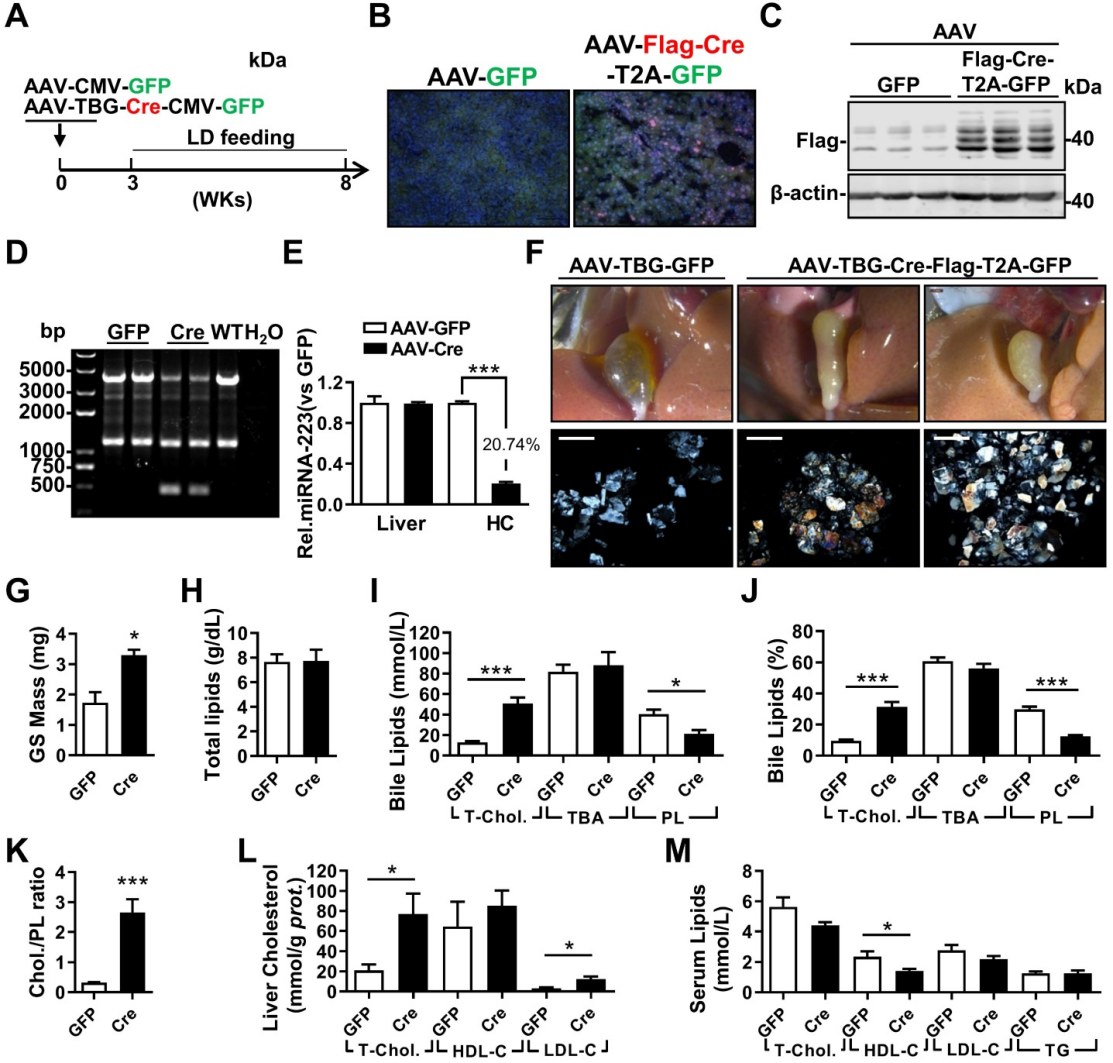
** The influences of hepatocytes specific miRNA-223 KD on mice gallstone formation. (A)** Hepatic specific miRNA-223 KD were conducted by one time *i.v* injection of with AAV-TBG-Flag-Cre or AAV-TBG-GFP (1×10^11^ virus genome) to male miRNA-223 cKO mice and 3 weeks later, the animals were feed with LD for additional 5 weeks, thereafter serum, livers and bile were harvested for further analysis. **(B)** Hepatic Flag-Cre overexpression were visualized by immunofluorescence against Flag tag in frozen liver sections (Flag, red; GFP, Green; DAPI, blue. Scale bars, 100 µm) and by **(C)** western blotting (~42 kDa for Flag-Cre); The miRNA-223 KD efficacy was further determined by **(D)** hepatic genome excise (cKO:4130bp, KD: 458bp); **(E)** miRNA-223 expression in livers and HCs determined by RT-qPCR (n=4-6 mice per group). **(F)** Representative images showing the gall bladders and cholesterol crystals in bile. Scale bars, 1 mm (gall bladder images) and 250 µm (cholesterol crystal images); **(G)** gallstone mass; **(H)** total bile lipids contents; **(I & J)** bile lipids content and percentage; **(K)** ratio of cholesterol to PL in bile (n=6-8 mice per group); biochemistry parameters of Cholesterol, HDL-C, LDL-C or TG were separately determined in **(L)** serum and **(M)** liver tissues (n=6-8 mice per group). *p<0.05; **p<0.005; ***p<0.001versus GFP.

To exclude potential compensatory effects from genetic depletion strategy, adenovirus associated virus (AAV)-mediated hepatocytes-specific knockdown (KD) of miRNA-223 expression (miRNA-223^KDHep^, KD_Hepa_) was performed by using Flag-tagged Cre recombinase under control of TBG promoter. Three weeks after AAV-Cre infection, cKO mice was feed with LD for another 5 weeks (Figure 4A). The expression efficiency of Cre recombinase was visualized by immunofluorescent staining against Flag tag (Figure 4B) and further verified by western blotting (Figure 4C). Consequently, we detected liver genomic DNA excision for miRNA-223 loci (Figure 4D) and an approximately 79.26% reduced miRNA-223 expression in freshly isolated hepatocytes (Figure 4E), although the general miRNA-223 amount in livers was not changed (Figure 4E). Consistently, KD_Hepa_ mice displayed similar phenotypes (*e.g* gallstone promoting effects and lipids content changes) which has been seen in ΔHepa mice (Figure 3F-K). Moreover, in KD_Hepa_ mice, serum HDL-C levels were slightly reduced (Figure 4L) and liver T-Chol., LDL-C and TG were significantly enhanced (Figure 4M, Supplementary Figure 4F). Similar to ΔHepa, KD_Hepa_ would not cause further liver injury and leukocytes infiltration (Supplementary Figure 4B & D).

Next, we examined the effects of miRNA-223 KD_Hepa_ on bile secretion via a modified bile duct catheration method. As indicated in Supplementary Figure 5A & B, miRNA-223 KD_Hepa_ was not able to affect bile secretion speed both in chow or LD feeding conditions. Meanwhile, bile total cholesterol level in KD_Hepa_ mice was significantly elevated after LD treatment (Supplementary Figure 5C), but levels of PL and TBA was not affected (Supplementary Figure 5D & E). Furthermore, Fourier Infra-red Spectrograph found gallstones from KD_Hepa_ mice showing stronger transmission rate drop at cholesterol specific absorption wavelengths (Supplementary Figure 5F), demonstrating higher cholesterol contents in the gallstones.

### miRNA-223 Targets Key Transporters of Biliary Cholesterol Secretion Pathway

**Figure 5:**
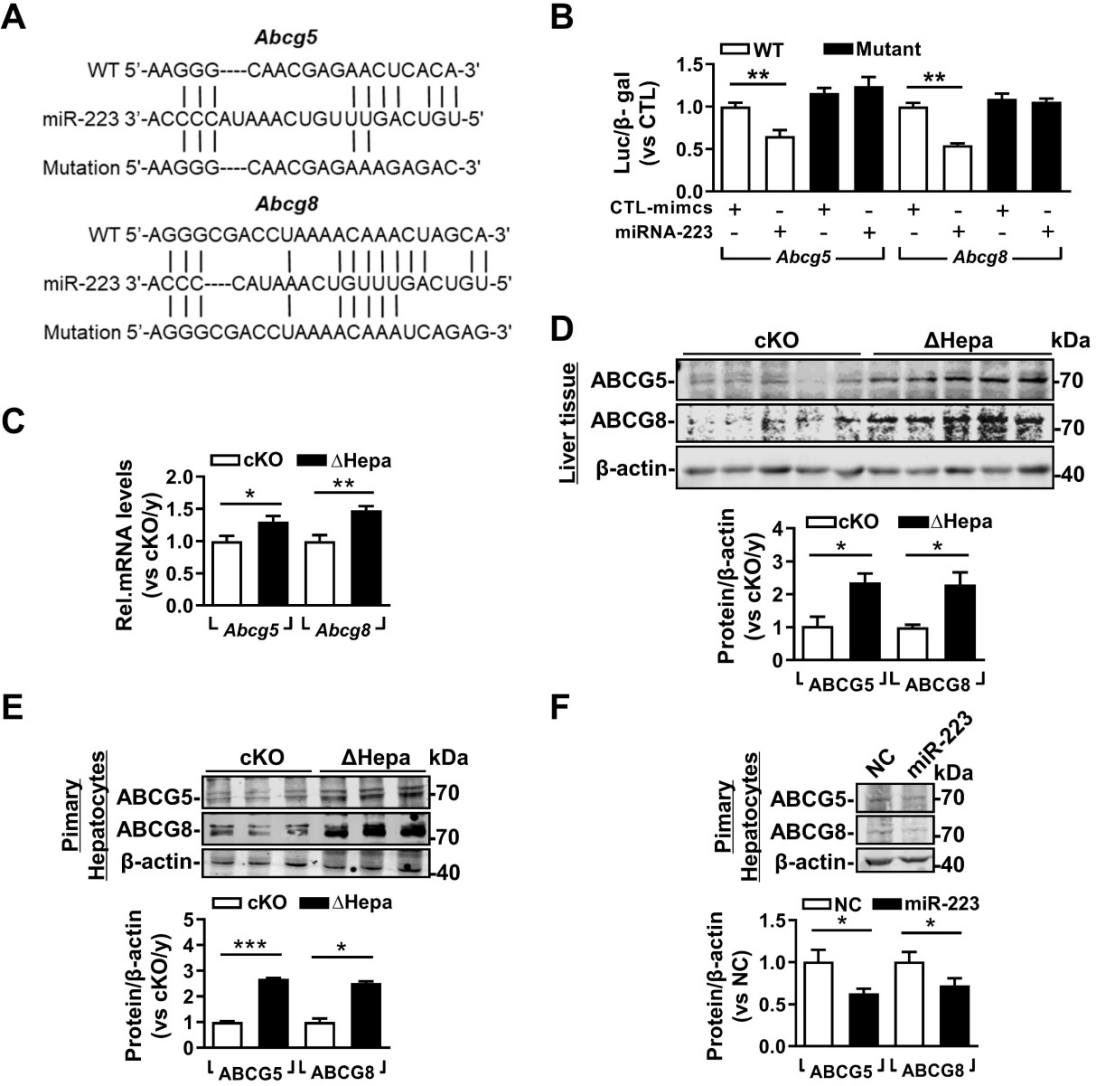
** miRNA-223 targets *Abcg5* and* Abcg8* expression in murine hepatocytes. (A)** Predicted binding sequences of miRNA-223 in 3'UTR regions of mouse Abcg5 and Abcg8 mRNA and **(B)** the effects of miRNA-223 mimics overexpression on luciferase activities determined from wild type or muated forms 3'UTR reporter gene assays (n=3 time indepemdent expreiments). With 5 weeks LD feeding, hepatic **(C)** mRNA and **(D)** protein expression levels of ABCG5 and ABCG8 in ΔHepa and cKO livers were analysed by mRNA by RT-qPCR and Western blotting (n=5 mice each group). **(E)** Protein levels of ABCG5 and ABCG8 from freshly isolated primary hepatocytes were examined by Westen blotting (n=3 mice per group). **(F)** Western blotting showing the protein levels of ABCG5 and ABCG8 from cultured primary hepatocytes transfected with miRNA-223 mimics or control mimics (n=3 independent experiments). *p<0.05; **p<0.005; ***p<0.001 versus cKO or control.

Based on the high concentration of biliary cholesterol secreted from hepatic miRNA-223 deficient mice, we postulated that miRNA-223 might affect biliary cholesterol transportation. Indeed, we found potential miRNA-223 binding sequences on the 3'untranslational region (UTR) of murine *Abcg5* and *Abcg8* mRNAs, the major transporters for billary cholesterol secretion (Figure 5A). By using 3'UTR reporter gene assays, overexpression of miRNA-223 mimics was readily suppressing their WT reporter gene activities and the effects were eliminated when the seeding sequences were mutated (Figure 5B). Furthermore, we verifed the both mRNA and protein expression levels of ABCG5 and ABCG8 were all significantly increased in ΔHepa livers (Figure 5C & D) or primary hepatocytes (Figure 5E) in compare with that in cKO controls. Moreover, miRNA-223 mimics transfection would decrease ABCG5 and ABCG8 proteins expression in cultured primary mouse hepatocytes (Figure 5F).

**Figure 6:**
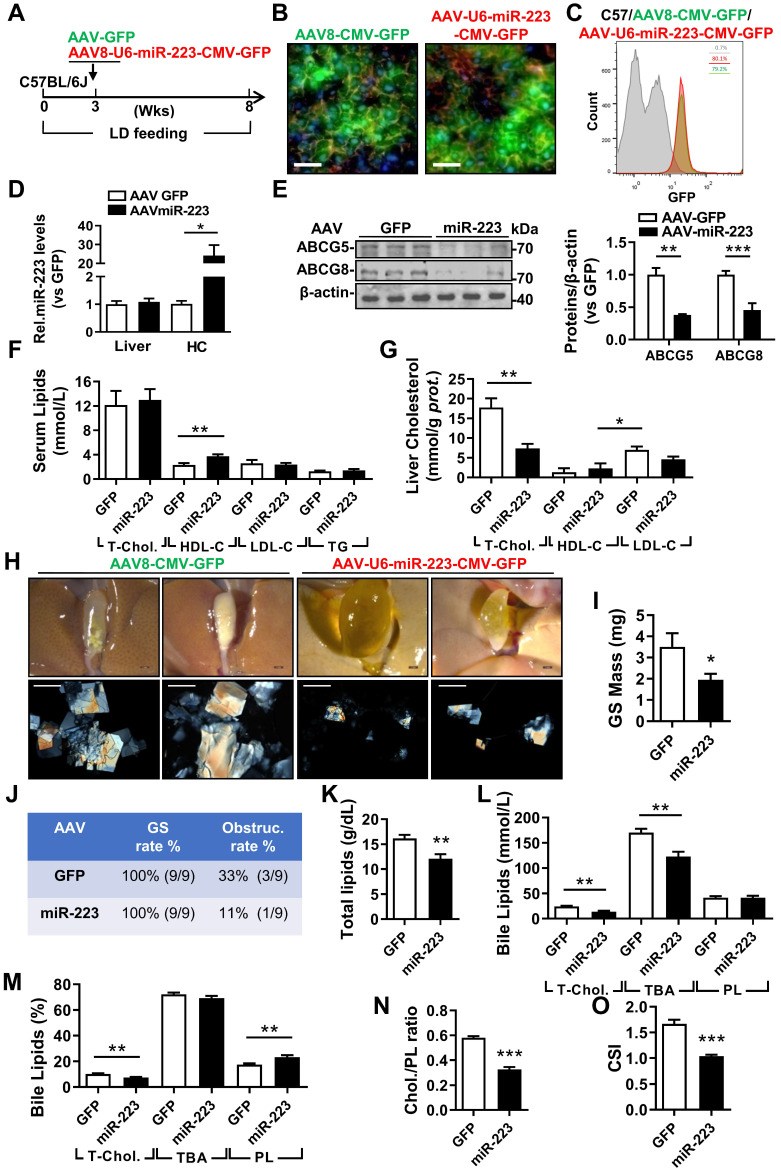
** The therapeutic attempt against mice gallstone progression by specific OE of miRNA-223 in hepatocytes.** WT mice were pretreated with LD for 3 weeks followed by (**A**) one-time injection with AAV8-U6-miRNA-223/CMV-GFP or AAV8-CMV-GFP (1×10^11^ virus genome) and continued LD feeding for additional 5 weeks, thereafter serum, livers and bile were harvested in the indicated time points for further analysis. AAV infection efficiency was assessed by (**B**) IF staining from frozen liver sections (GFP, green; DAPI, blue; F-actin, red. Scale bars, 50 µm) as well as by (**C**) FACS for GFP positive cells population from freshly isolated primary hepatocytes. **(D)** RT-qPCR evaluated miRNA-223 OE efficiency in liver and primary hepatocytes (n=3-5 mice per group); (E) the protein expression of ABCG5 and ABCG8 were examined in lives by Western blotting (n=3 mice per group); biochemistry parameters of total cholesterol, HDL-C, LDL-C and TG were determined in (**F**) serum and (**G**) liver tissues (n=9 mice per group). **(H)** Representative images showing the gall bladders and cholesterol crystals in bile. Scale bars, 1 mm (gall bladder images) and 250 µm (cholesterol crystal images). **(I)** Weighted gallstone mass (n=9 mice per group). **(J)** Table summarized gallstone formation rate and obstruction rate; (**K**) total bile lipids contents; (**L & M**) bile lipids contents and percentage; (**N**) ratio of cholesterol to PL as well as (**O**) cholesterol saturation index (CSI) (n=6-8 mice per group). *p<0.05; **p<0.005; ***p<0.001 versus GFP.

As a previous study demonstrated miRNA-223 KO unbalanced hepatic/blood cholesterol homeostasis via regulating a series of targets genes expression in cholesterol synthesis (*Hmgcs*, *Acat2 and Sc4mcl*), uptake (*Scarb1*) and efflux (*Abca1*) 13, we also checked those genes expression in KO and ΔHepa livers. For cholesterol sythesis related targets, increased mRNA expression of *Hmgcs* and *Acat2* were only detected in ΔHepa livers, however the Hmgcs protein expression was not changed (Supplymentary Figure 6A&B). Meanwhile, *Sc4mcl* seems not to be linked with miRNA-223 as its mRNA expression was comparable in KO and ΔHepa livers with their control livers (Supplymentary Figure 6A). Moreover, *Scarb1* was shown to be upregualted in protein levels in ΔHepa livers and primary hepatocytes with no changes in mRNA expression. Besides, consistant increases in both mRNA and protein levels of Abca1 were found in ΔHepa livers (Supplymentary Figure 6A & B).

### miRNA-223 Overexpression in Hepatocytes Prevents LD-induced Gallstone Progression

**Figure 7:**
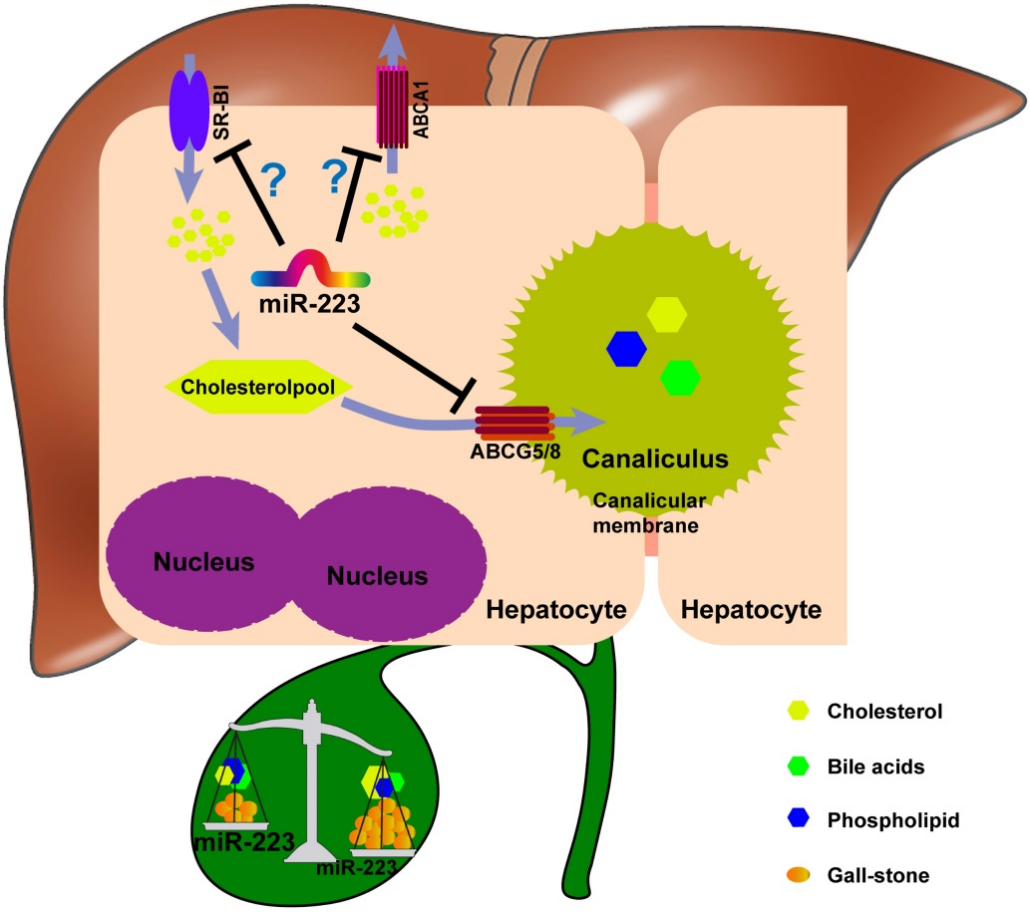
The potent role of miRNA-223 in regulating mice gallstone development.

To evaluate the therapeutic potential against gallstone progression by specificly increasing miRNA-223 levels in hepatocytes, administration of AAV8-U6-miRNA-223/CMV-GFP (OE) or AAV8-CMV-GFP (GFP) towards WT mice was conducted in the third week during an eight-weeks LD feeding frame (Figure 6A). The AAV infection rates were comparable as determined by GFP signals from liver frozen sectionsand ~80% GFP positive primary hepatocytes in both groups by FACS analysis (Figure 6C). Consequently, approximately 20-fold increased miRNA-223 levels was detected in primary hepatocytes isolated from miRNA-223 OE mice Figure 6D), leading to attenuated hepatic protein expression in ABCG5, and ABCG8(Figure 6E), though their mRNA was not intensively affected (Supplymentary Figure 7A). Additionally, reduced protein expression of SR-BI and ABCA1 were also detected in primary hepatocytes isolated from miRNA-223 OE livers (Supplymentary Figure 7B). Moreover, as serum lipids analysis indicated (Figure 6F), except slight increased HDL-C levels, miRNA-223 OE would not affect other serum lipids levels. However, remarkable reducd contents of T-Chol. LDL-C and TG were observed in miRNA-223 OE liver tissues (Figure 6G, Supplymentary Figure 7E). Importantly, miRNA-223 OE would partially prevent further gallstone progression as evidenced by decreased appearance of the gallbladder stones (Figure 6H), lower gallbladder obstruction rate (Figure 6J, 11.1% in OE *v.s* 33% in GFP group) as well as reduced gallstone mass (Figure 6I). Moreover, miRNA-223 OE reduced total biliary lipids content (Figure 6K), T-Chol. and TBA levels (Figure 6I & L). Further bile lipids composition calculation revealed there are relative lower percentage of cholesterol and higher percentage of PL in miRNA-223 OE bile (Figure 6M), leading to a declined cholesterol/PL ratio (Figure 6N) and attenuated cholesterol saturation index (CSI, shown in Figure 6O). Besides, AAV-miRNA-223 OE would not induce further liver damage as indicated by the similar ALT and AST activities in serum in comparion with GFP mice (Supplementary Figure 7C & D).

Taken together, we proposed a novel mechanistic role of miRNA-223 in regulating LD-induced cholesterol gallstone development (Figure 7): miRNA-223 preferentially decreases cholesterol transportation from hepatocytes into the bile by directly targeting ABCG5 and ABCG8 at the canalicular membrane. The blockage effects of miRNA-223 on cholesterol biliary secretion pathway give rise to attenuated bile cholesterol content and saturation status, consequently prevent cholesterol gallstone formation. Besides, basolateral cholesterol transporters SR-BI and ABCA1 mediate cholesterol transportation between blood and hepatocytes and they are also targeted by miRNA-223 via indirect mechanisms, might contributing total cholesterol homestasis in blood.

## Discussion

The purpose of this study was to determine the importance of hepatocytes miRNA-223 in regulating cholesterol biliary secretion and gallstone formation by using a series of genetically modified mouse models. The major finding is that miRNA-223 plays a pivotal role in regulating hepatobiliary cholesterol secretion and cholesterol gallstone formation by targeting key cholesterol transporters.

We found that the LD with 1.25% cholesterol readily increases miRNA-223 expression in mouse livers. However, the increased miRNA-223 expression in liver tissue would be mainly attributed to infiltrated myeloid cells or Lyz-2 positive nonparenchymal cells because there was a 78% reduction of miRNA-223 expression in livers from myeloid specific miRNA-223 KO mice. Due to relative lower content of miRNA-223 in hepatocytes, we suggested liver miRNA-223 alternation would not reflect the issues occurred in hepatocytes. Therefore, we prepared primary hepatocytes and determined miRNA-223 expression was significantly increased in freshly isolated primary hepatocytes from LD treated animals. The data were consistent with previous *in vitro* studies, in which cholesterol deprivation would time-dependently suppress miRNA-223 expresison in cultured human hepatocytes 13. Besides, a recent study reported that high fat diet feeding would increase miRNA-223 expression in mouse liver and hepatocytes 26. Additionally, we observed LD mildly induces hepatitis and fibrosis in miRNA-223 KO,while such abnormalities were not appeared in hepatocyte-specific miRNA-223 KO (ΔHepa) mice, suggesting that hepatocytes miRNA-223 is not involved in the development of pathological changes at least in current experimental settings.

To better determine the role of liver miRNA-223 in cholesterol gallstone formation, we generated a conditional miRNA-223 KO (cKO) mice model and obtained conventional (KO), hepatocytes-specific (ΔHepa), and myeloid-specific KO (ΔMye) mice by crossbreeding with EIIA-Cre, Alb-Cre, and Lyz-Cre transgenic mice. We also performed AAV-TBG-Cre-mediated hepatocytes-specific knockdown (KD_Hep_) in miRNA-223 cKO mice. By phenotypic analysis, LD feeding promoted gallstone development in KO, ΔHep and KD_Hep_ mice but not in ΔMye mice, fully supporting the importance of hepatocytes miRNA-223 in regulating hepatic/biliary cholesterol homeostasis. Besides, we noticed miRNA-223 expression levels were correspondingly decreased or increased in primary hepatocytes isolated from ΔHep, KD_Hep_ or OE mice, however unchanged miRNA-223 levels were observed in liver tissues from those mice (Figure 3A, 4E, and 6D), which would be explained by the lower expression levels of miRNA-223 in hepatocyte and the contribution of hepatocytes to general hepatic miRNA-223 levels is as less as 0.07% (shown in Figure 1B), therefore, neither loss nor gain of miRNA-223 expression in hepatocytes would be unable to obviously influence miRNA-223 expression in liver tissue.

Interestingly, a recent study reported “extracellular vesicles mediated miRNA-223 transfer from myeloid cells to hepatocyte” in mice NASH model 27. It is an important discovery showing the possibility that hepatocyte miRNA-223 levels would be regulated or derived from different cell types in certain pathological conditions. We deduces the determinate point for functional “transfer” should be attributed by the amount of hepatic infiltrating myeloid cells, which further facilitate miRNA-223 containing vehicles release and transfer into injured hepatocytes, eventually regulates hepatocyte function. In the NASH model, long term (at least 3 month) HFD treatment remarkably induces liver toxicity (higher ALT and AST levels) and further recruit intensive number of myeloid cells infiltrating into liver tissue, the severe pathological challenges provide a proper space for the “extracellular transfer”. In compare with that, the mouse gallstone model induced by lithogenic diet containing mild high fat diet (1.25% cholesterol, 15% fat) with short term (1-5 weeks) feeding is unable to cause obvious pathological changes in liver morphology and liver injuries (the similar serum AST and ALT values as chow diet treatment). We also found lithogenic diet treatment would slightly increase myeloid cell infiltrating into liver, it suggests a possibility for the “miRNA-223 containing vehicles transfer” into hepatocytes. However, it seemed the “transfer” contribute less to mice gallstone development as we did not detect obvious differences in major gallstone phenotypes between myeloid miRNA-223 KO mice and control littermates cKO mice, which would be possibly explained by the less extent of hepatic infiltrating myeloid cells, even if limited intracellular vehicles transfer occurred, it would not efficiently affect the whole liver cholesterol metabolism.

Although the lower expression level, hepatocyte expressing miRNA-223 was previously reported to play important roles in hepatic biological synthesis, cholesterol uptake, and efflux by targeting a complex gene set including *Hmgcs1*,* Sc4mcl*, *Acat2*,* Scarb1and Abca1 etc.,* and miRNA-223 KO contributes to hypercholesterolemia and augments cholesterol accumulation in the liver 13. However, our data would only partial identical with their major findings. Similarly, Our data would also confirm the higher cholestrerol contents in livers from miRNA-223 KO, ΔHepa and KD mice, however the expression of previously identified cholesterol synthesis related target genes (*Hmgcs1*,* Sc4mcl* and *Acat2*) were all shown to be less correlation with miRNA-223, suggesting other targets or mechanism might contribute to the phenotype. Inconsistantly, neither miRNA-223 KO nor ΔHepa and KD would support the links between miRNA-223 deficency with hypercholesterolemia. Of the note, upregulation of SR-BI and down regulation of ABCA1 in human hepatocyte cell line was addressed to be involved in miRNA-223 mediated hepatocyte cholesterol uptake and efflux 13, however our *in vivo* data from miRNA-223 ΔHepa and OE mice proved the both were all negatively regulated by miRNA-223, herein balancing cholesterol exchanges between liver and blood. Our current study focused on *in vivo* functional identidication by using hepatocyte specific loss or gain expression of miRNA-223 to dessect its role in regulating liver cholesterol transportation in mice. The differences of our fingdings with previous study might be reasoned by different experimental settings such as genetic modified animals, animal modles or species *etc.* Although inconsistence exisited, our data and previous study all strongly support the important role of hepatocytes miRNA-223 in regulating cholesterols balance. Based on consisitant higher cholesterol contents in liver and less influenced serum cholesterol levels in miRNA-223 KO, ΔHepa and KD livers, we wonder how to process the excess cholesterol in miRNA-223 deficent livers. Our data further reported hepatocyte specific loss of miRNA-223 expression greatly promote cholesterol gallstone formation, revealing a completely new function of miRNA-223 in hepatic cholesterol homeostasis.

Our first novel finding is that miRNA-223 in hepatocytes plays a pivotal role in controlling biliary cholesterol secretion by direct targeting the canalicular cholesterol transporters ABCG5 and ABCG8. ABCG5/8 has been found to contribute ~75% biliary cholesterol secretion, and its genetic mutation, or dysregulation would strongly disturbs cholesterol secretion and cholelithiasis progression. Our data solidly demonstrate that in LD feeding condition distrupting miRNA-223 expression in hepatocytes would significantly increases ABCG5 and ABCG8 expression and consequently elevates cholesterol levels in both liver secreted and bladder storaged bile. Meanwhile, we also detected increased SR-BI protein expression, which is believed to be a ABCG5/8 independent cholesterol transporter localized in canalicular membranes and conducting biliary cholesterol secretion in physical condtion 7, while its exact role in gallstone development remains unclear. Of the note, significant increased SR-BI expression evidenced in miRNA-223 hepatocytes specific KO livers, which is opposite with the previous reports in which reduced SR-BI expression appeared in miRNA-223 KO livers. The inconsistent observation perhaps derived from different strategys to generate miRNA-223 loss of functional mice or the specific experimental models.

Reversely, AAV-mediated hepatocytes-specific miRNA-223 OE in WT mice reduces expression of ABCG5, ABCG8 and decreases cholesterol content in the bile. In current study, we demonstrated a negative regulation manner between miRNA-223 and those cholesterol transporters in mouse hepatocyte. Although not noted in current available miRNA targets prediction databases, imperfect binding sequences of miRNA-223 were found in 3'UTR regions of *Abcg5* and *Abcg8* mRNA, suggesting a possible directly regulating manner. Therefore, hypothsis was experimental confirmed by classical 3'UTR reporter assays.

To our knowledge, conventional miRNA-223 KO mice were wildly used in various research fields, however, the tissue or cell type specific miRNA-223 KO animal is emergently required for precisely verifying its functional importance and molecular mechanism in interested cell types or tissues. Therein, we generated miRNA-223 KO, hepatocyte specific miRNA-223 KO and KD mice, demonstrating importance of hepatocyte expressing miRNA-223 in regulating gallstone development via targeting cholesterol transporters. Meanwhile, we also suggested certain retrospective studies should be considerd by using cell specific miRNA-223 KO mice, which whould be able to enrich, verify or correct our current knowledges and understandings towards miRNA-223 biological functions.

Our second important finding is the novel role of miRNA-223 in cholesterol gallstone formation. Gallstone crystallization occurs in supersaturated bile where BA and PL cannot dissolve excessive cholesterol. Supersaturated bile could be attributed by 1) hyper-cholesterol secretion; 2) reduced biliary BA or PL secretion with unaffected cholesterol secretion; 3) increased cholesterol and reduced BA or PL secretion 3. Our study suggested that accelerated gallstone formation in hepatic miRNA-223 KO/KD mice is strongly associated with supersaturated cholesterol status mainly attributed by an excess of cholesterol deposition in bile.

More interestingly, insufficient biliary PL levels were also determined in miRNA-223 deficient mice that would further decline cholesterol solubility, which also caused our interest. ABCG4 is known as a main PL transporter faciliateing hepatic PL secretion towards bile and its mRNA expression level was slightly increased in miRNA-223 deficent livers, it would be explained by a negative feedback way in which insufficient PL storage in bile requires more ABCG4 to increase biliary PL transportation. Similary, increased BA transporter ABCG11 in miRNA-223 deficent livers might attempt to balance the oversaturated cholesterol status in bile. It is a complexed gene regulating network for PL and BA metabolism in hepatocytes and more detailed works needed in the future studies to determine the potent role of miRNA-223 in regulating hepatic PL and BA homeostasis.

Besides, recent research also indicates that gallbladder motility insufficiency is greatly affected by the loss of interstitial Cajal-like cells and telocytes that further contributes to the cholelithiasis 28, 29. Apparently, whether hepatic miRNA-223-affected bile saturation might have association with gallbladder motility dysfunction (especially Cajal-like cells and telocytesloss) is an interesting question and worthwhile to explore in the future study.

Lastly, this study provides the evidence for the miRNA-223 to be an effective therapeutic target in cholesterol gallstone disease. To evaluate the therapeutic efficacy by manipulating miRNA-223 expression, we conducted AAV-mediated miRNA-223 overexpression in WT mouse liver. The results demonstrated the strategy would efficiently decrease bile cholesterol content and reduced gallstone formation, supporting a novel therapeutic strategy for cholesterol gallstone disease by targeting miRNA-223.

In summary, the present study uncovers an important role of miRNA-223 in regulating hepatobiliary cholesterol secretion and cholesterol gallstone formation by targeting key cholesterol transporters ABCG5 and ABCG8 in cholesterol biliary secretion pathway. Proof-of-concept study using AAV-mediated miRNA-223 OE in WT mouse liver shows promising therapeutic efficacy in treating LD-induced cholesterol gallstone. Hence, miRNA-223 has been identified as a novel potential therapeutic target in cholesterol gallstone disease.

## Supplementary Material

Supplementary figures and tables.

## Abbreviations

miRNA-223: microRNA-223
LD: lithogenic diet
ABCG: ATP-binding cassette sub-family G
T-Chol.: total cholesterol
BA: bile salt
PL: phospholipids
TG: triglyceride
BM: bone marrow
