# Supplementary material for: miRNA-223 Suppresses Mouse Gallstone Formation by Targeting Key Transporters in Hepatobiliary Cholesterol Secretion Pathway
Source: Int J Biol Sci. 2021 Oct 25;17(15):4459–73. doi: 10.7150/ijbs.65485 (PMC8579439; doi:10.7150/ijbs.65485)
Supplement: Supplementary file 1 — Supplementary figures and tables. [file ijbsv17p4459s1.pdf]

Supplementary Figures:

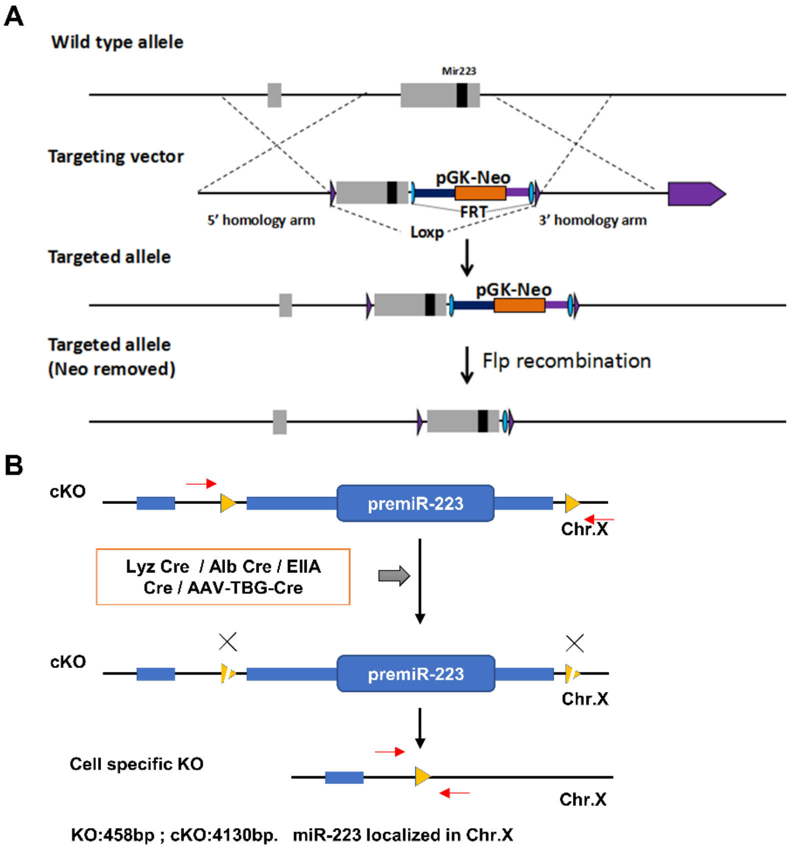

Supplementary Figure 1. Strategys of generating miRNA-223 conditional knockout and cell specific knockout / knockdown mice stains. (A) miRNA-223(ENSMUST00000102112) was localized in the second Exon of ncRNA F630028O10Rik, two Flox sequences were designed in the Intron 1 and down stream of Exon 2. By homologue recombinant targeting principle in embryonic stem cells, targeting vector were prepared containing 9.616 kb 5' homologue arm, 3.566 kb Flox region, PGK-Neo-poly A region flanked with FRTs, 4.919 kb 3' homologue arm, and MC1-TK-poly A negative selection region. The positive EC clones were obtained by G418 and Ganc selection and genomic DNA sequencing verification, and chimeric offsprings were obtained after implantation of C57BL/6J blastaea injected with recombinant ES cells into surrogate mice. Neo cassette was removed by breeding chimeric mice with Flp^{Tg} mice. All founder mice were verified by genomic DNA sequencing; (B) Scheme showing the stragte for generating tissue or cell specific miRNA-223 KO mice by crossbreeding miRNA-223 cKO mice with indicated Cre mice or virus infection.

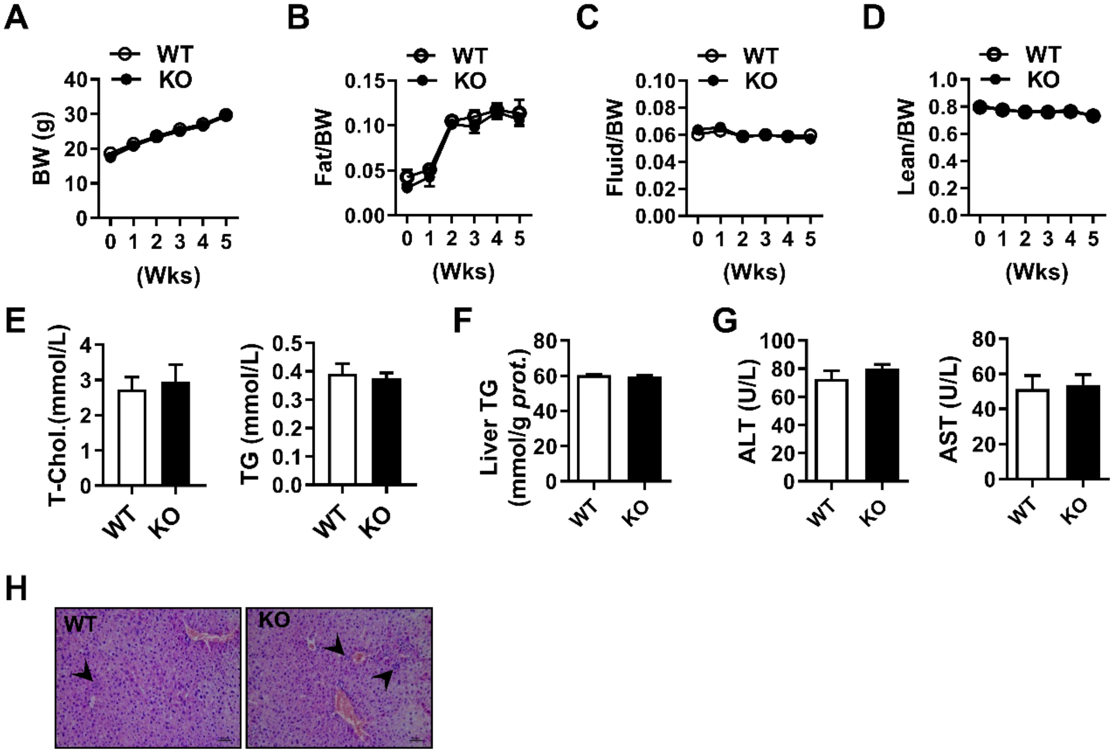

Supplementary Figure 2. During 5 weeks LD treatment, (A) body weight changes, (B) Fat contents, (C) Fluid contents and (D) Lean contents were evaluated in the indicated time for in WT and KO mice (n=5 mice each group). After 5 weeks LD feeding, WT and KO mice liver tissues were collected for (E) TG contents; (F) ALT and AST activities in serum were determined, (G) H&E staining for liver sections and arrow indicated the infiltrating inflamatory cells. Scale bars, 50 μm.

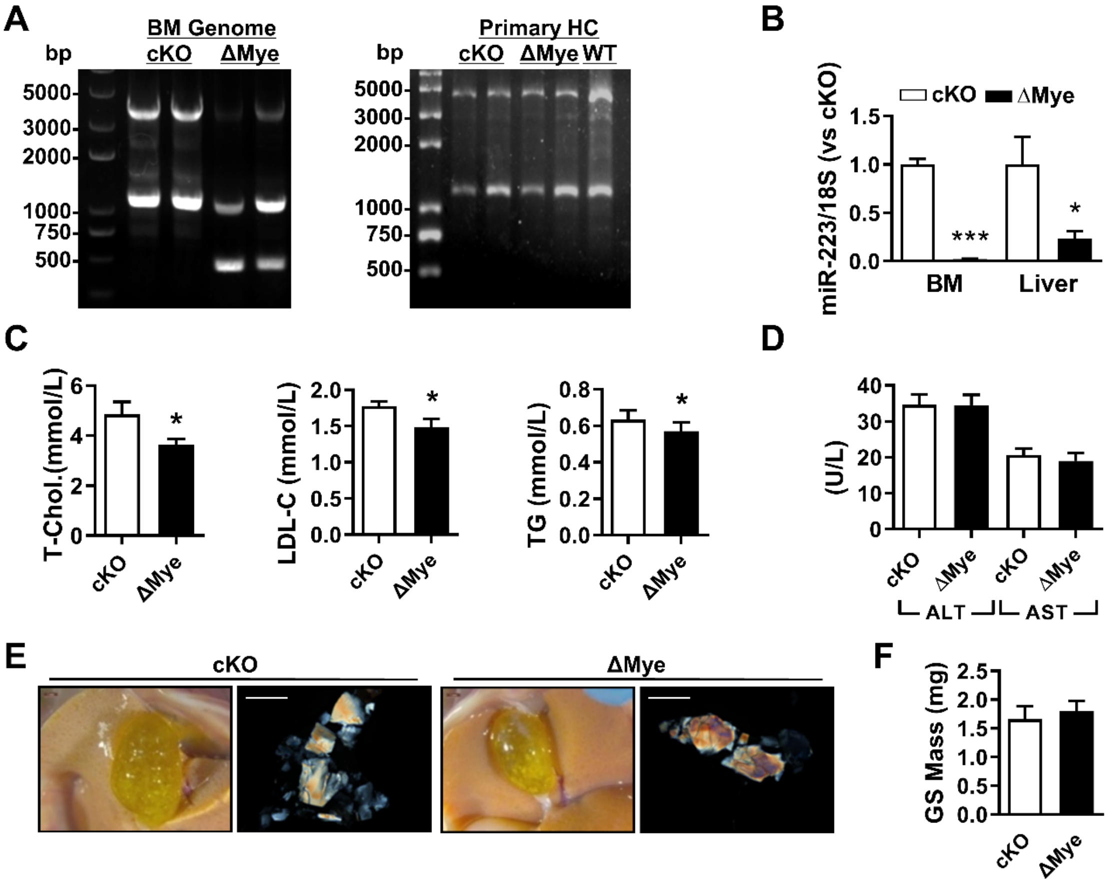

Supplementary Figure 3 (A) PCR showing the genomic DNA precision for Bone marrow (BM) and primary hepatocytes (HC), and (B) RT-qPCR revealed miRNA-223 epxression levels in BM and HC from miRNA-223 KO mice (Δmyeloid/y) and cKO mice (cKO:4130bp, KO:458bp, n=4 mice each group); With 5 weeks LD treatment, thelevels of (C) Total-Cholesterol , LDL-C and TG in livers and (D) Serum ALT and AST acitivities were determined (n=10 each group); (E&F) gallstone phenotype and gallstone mass were further determined (n=4 each group). **p*<0.05; ****p*<0.001 versus cKO. Scale bars, 1 mm (gall bladder images) and 250 µm (cholesterol crystal images).

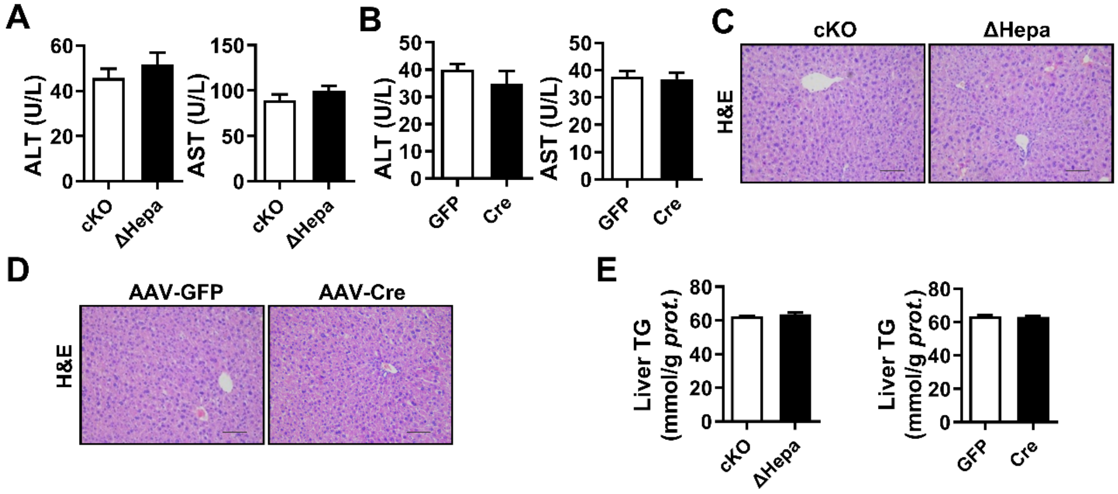

Supplementary Figure 4. The effects of hepatocute specific miRNA-223 KO or KD on liver injury and TG livers. ΔHepa and cKO mice (n=10 mice each group) or AAV-TBG-Cre and AAV-GFP treated mice (n=6-8 mice each group) were fed with LD for 5 weeks, serum were used to determined (A&B) ALT and AST activities, liver tissues were analyzed for (C&D) H&E staining and (E) TG contents. Scale bars, 100 μm.

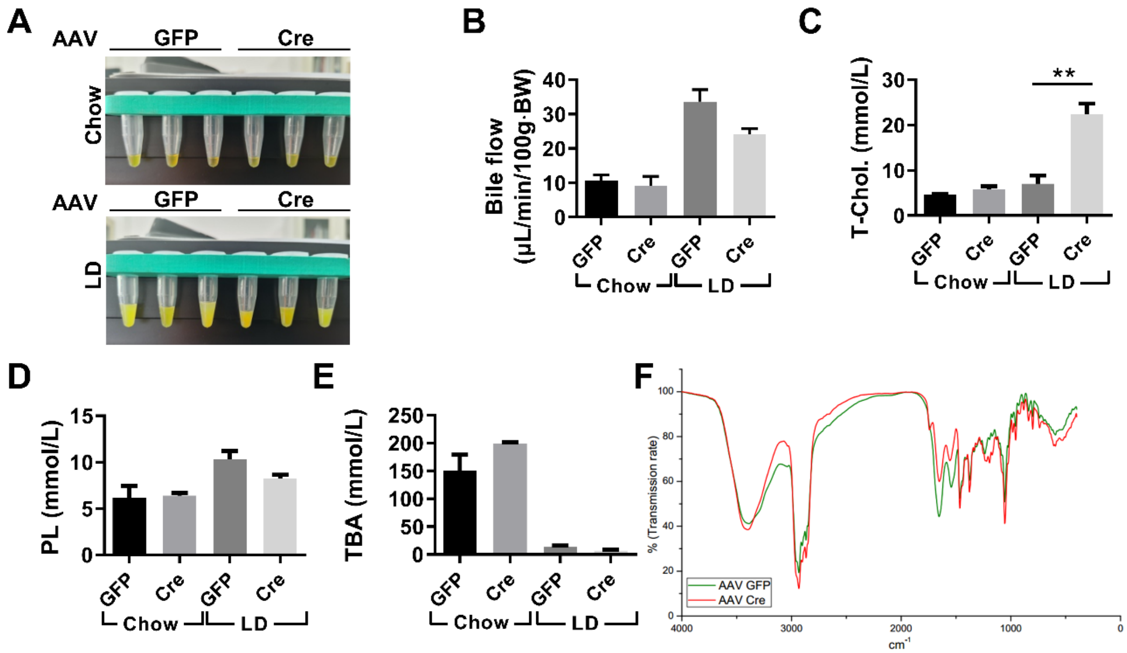

Supplementary Figure 5. The effects of miRNA-223 knockdown on mice bile secretion and lipid contents in bile and gallstone. Hepatocyte specific miRNA-223 knockdown were conducted via AAV8-TGB-Cre or AAV8-CMV-Cre (control) *i.v.* injection for miRNA-223 cKO mice and three weeks later, those mice were fed with chow or LD for additional three weeks. Bile flow rate was determined via catheterization with PE-10 tube within 30 min. (A) representative image showing the liver secreted bile volume; (B) Bar graph showing bile flow rate Bile lipids content of (C) T-Cholesterol, (D) PL and (E) TBA were separately determined from liver secreted bile. Data are summarized from 3-4 mice each group and **p<0.01 versus GFP. (F) The affects of miRNA-223 KD on cholesterol contents in mice gallstone. Gallstones were collected and mixed with KBr (1:100) and further prepared for Fourier Infra-red Spectrograph analysis. The cholesterol specific regions are at 2960.244, 2935.173, 2902.383, 2867.676 cm^{-1}. Gallstones from 3 mice each group were mixed and subjected to Fourier Infra-red Spectrograph analysis.

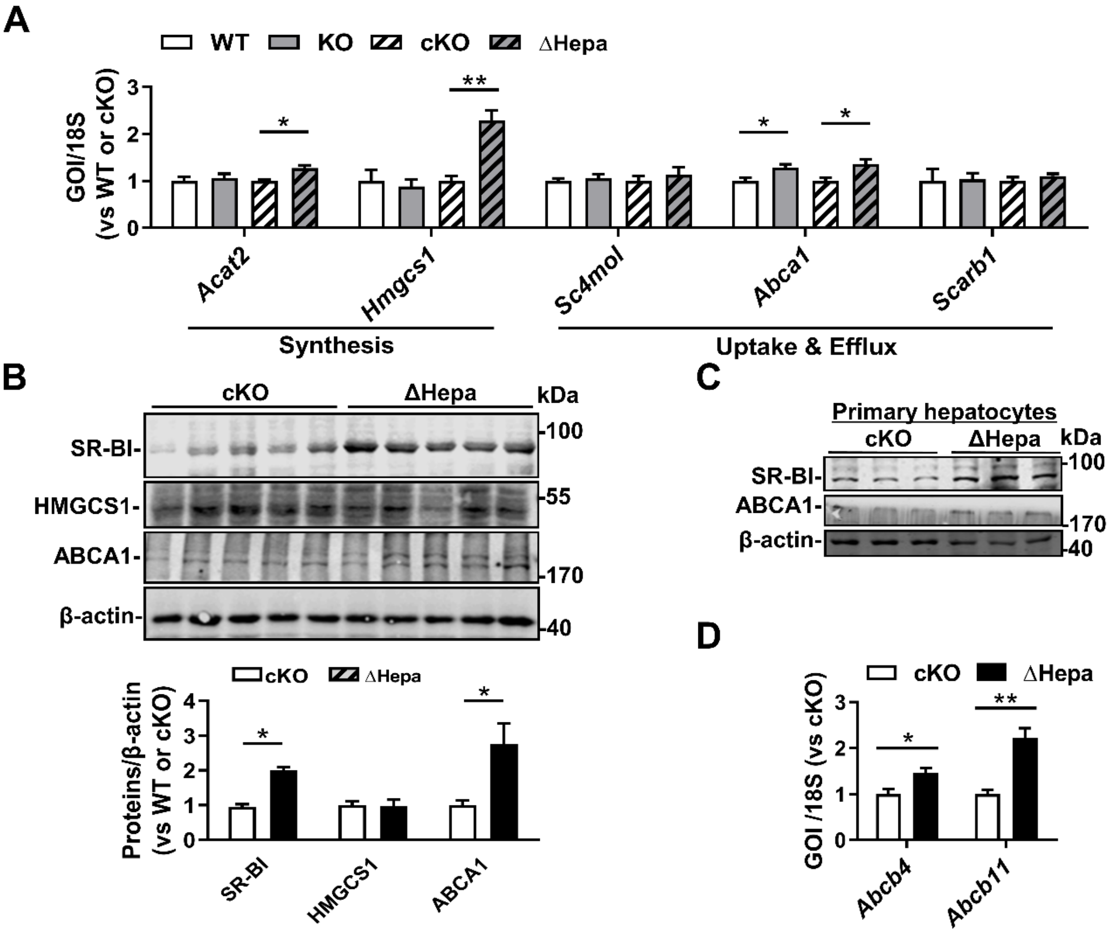

Supplementary Figure 6. The gene expression was determined by RT-qPCR or western blotting in livers samples from KO and WT or ΔHepa and cKO mice with LD challenge for 5 weeks. (A) RT-qPCR assessed the selected genes expression concerning hepatic cholesterol synthesis, uptake and efflux as well as biliary secretion (n=5-6 mice per group). (B) Liver protein levels for SR-BI, HMGCS1 and ABCA1 as well as (C) primary hepatocytes expressing SR-BI and ABCA1 were determined by Western blotting (n=3-5 mice per group). (D) RT-qPCR exams the mRNA expression for indicated genes from livers of cKO and ΔHepa mice (n=5-6 mice per group). *p<0.05, **p<0.01 versus WT or cKO.

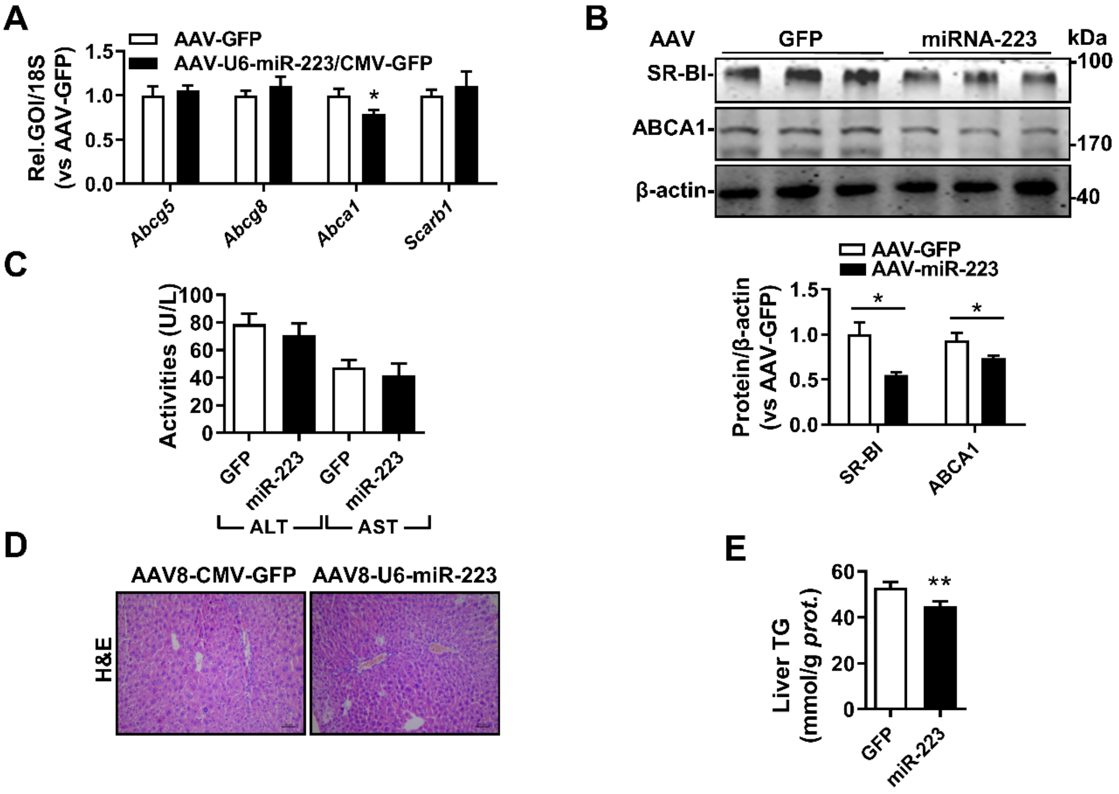

Supplementary Figure 7. Supplementary Figure 7. WT mice were pretreated with LD for 3 weeks followed by one time injection with AAV8-U6-miRNA-223/CMV-GFP or AAV8-CMV-GFP (10^{11} virus genome) and continued LD feeding for additional 5 weeks, (A) mRNA expression of *Abcg5*, *Abcg8*, *Abca1* and *Scarb1* were determined by RT-qPCR (n=5 mice per group); (B) protein levels of SR-BI and ABCA1 were detected by western blotting (n=3 mice per group); (C) serum ALT and AST activates (n=9 mice per group); (D) H&E staining for liver sections. Scale bars, 50 μm; (E) liver TG levels (n=9 mice per group).*p<0.05, **P<0.01 versus GFP.

Supplementary Tables:

Supplementary Table 1. Forward and Reverse Primers Used for RT-qPCR, Genotyping and Plasmid construction

Gene	Forward Primer (5´→3´)	Reverse Primer (5´→3´)	Products(bp)
18s	CTTTGGTCGCTCGCTCCTC	CTGACCGGGTTGGTTTTGAT	128
Abca1	GCTCTCAGGTGGGATGCAG	GGCTCGTCCAGAATGACAAC	81
Abcb11	CTCACAAAGAAACAGGCATAAAGG	GTTGACGGATGGAAGCTCTTA	111
Abcb4	TTGAAGTTGAGCTAAGTGACGA	GGCACGTTTGCATCAAGT	154
Abcg5	ATCTCTGGGCTGCTTATTG	AACTCATTGACCACGAGAATC	125
Abcg8	CACCTTTACACCACACAAATCG	TGACAATGAGGTAGATCGCATA	112
Acat2	ATTGTTGAAAGGTGGGCAGC	GGTAACATCCCATCCCGTCA	70
Hmgcs1	TGTGGTTCAGAAACTGATGG	TGTCTCCTGCAACTACCAGA	266
Sc4mol	TCCAGTTGCCTCTGATTTG	GGATGTGCGTATTCTGCTT	244
Scarb1	GCAAATTTGGCCTGTTTGTT	GATCTTGCTGAGTCCGTTCC	122
miRNA-223 KO	GCTGAAACAGTGCCCACAACAG	CACCCCAGTGCAATGATAGAATAT	
miRNA-223 cKO	CAAATACCAACCAGGGTTTTGC	TCCCTCCGACAATTCTGAGCAA	
Abcg5 3' UTR	AAGCTTAAACATAATTTTTAAATG	ACTAGTTTAAGATGACAGGCAGG	
Abcg8 3' UTR	AAGCTTGCTGAGACAACTGGATT	ACTAGTGTATGGAATGGGAACCA	
Abcg5 3' UTR Mut	CTGGAATAGCAGAGGGCGTGTCTTTCTCGTTGCC	AGACACGCCCTCTGCTATTCCAGCTTGTGGGGCA	
Abcg8 3' UTR Mut	ATAAGCGAAAAACATTTCTCTGATTTGTTTTAGG	TCAGAGAAATGTTTTTCGCTTATCTCTGCGTGGA	

Supplementary Table 2. Antibodies Used for Western Blot and FACS

Antibody	Biological source	Manufacturers (Locations)	Catalog no.
ABCG5	Rabbit	Proteintech (Wuhan, China)	27722-1-AP
ABCA1	Rabbit	Abclonal (Wuhan, China)	A7228
ABCG8	Rabbit	Abclonal (Wuhan, China)	A1880
beta Actin	Mouse	Abcam (Shanghai, China)	ab8226
FITC-Gr1	Rat	Biolegends (San Diego, CA)	108405
FITC-ISO	Rat	Biolegends (San Diego, CA)	102205
Flag	Rabbit	Proteintech (Wuhan, China)	20543-1-AP
HMGCS1	Rabbit	Proteintech (Wuhan, China)	17643-1-AP
PE-CD11b	Rat	Biolegends (San Diego, CA)	101207
PE-ISO	Rat	Biolegends (San Diego, CA)	400607
SR-BI	Rabbit	Abcam (Shanghai, China)	ab217318
